# A potential defensive role of TIM-3 on T lymphocytes in the inflammatory involvement of diabetic kidney disease

**DOI:** 10.3389/fimmu.2024.1365226

**Published:** 2024-05-15

**Authors:** Xiao-Jun Chen, Runyan Tang, Jie Zha, Li Zeng, Linshan Zhou, Zhiwen Liu, Danyi Yang, Mengru Zeng, Xuejing Zhu, Anqun Chen, Hong Liu, Huihui Chen, Guochun Chen

**Affiliations:** ^1^ Department of Nephrology, The Second Xiangya Hospital of Central South University, Changsha, China; ^2^ Department of Nephrology, Hunan Key Laboratory of Kidney Disease and Blood Purification, The Second Xiangya Hospital at Central South University, Changsha, China; ^3^ Department of Ophthalmology, The Second Xiangya Hospital of Central South University, Changsha, China

**Keywords:** diabetic kidney disease, T cell immunity, immune checkpoint molecules, TIM-3, inflammation, kidney homeostasis

## Abstract

**Objective:**

The aberrant mobilization and activation of various T lymphocyte subpopulations play a pivotal role in the pathogenesis of diabetic kidney disease (DKD), yet the regulatory mechanisms underlying these processes remain poorly understood. Our study is premised on the hypothesis that the dysregulation of immune checkpoint molecules on T lymphocytes disrupts kidney homeostasis, instigates pathological inflammation, and promotes DKD progression.

**Methods:**

A total of 360 adult patients with DKD were recruited for this study. The expression of immune checkpoint molecules on T lymphocytes was assessed by flow cytometry for peripheral blood and immunofluorescence staining for kidney tissue. Single-cell sequencing (scRNA-seq) data from the kidneys of DKD mouse model were analyzed.

**Results:**

Patients with DKD exhibited a reduction in the proportion of CD3+TIM-3+ T cells in circulation concurrent with the emergence of significant albuminuria and hematuria (p=0.008 and 0.02, respectively). Conversely, the incidence of infection during DKD progression correlated with an elevation of peripheral CD3+TIM-3+ T cells (p=0.01). Both univariate and multivariate logistic regression analysis revealed a significant inverse relationship between the proportion of peripheral CD3+TIM-3+ T cells and severe interstitial mononuclear infiltration (OR: 0.193, 95%CI: 0.040,0.926, p=0.04). Immunofluorescence assays demonstrated an increase of CD3+, TIM-3+ and CD3+TIM-3+ interstitial mononuclear cells in the kidneys of DKD patients as compared to patients diagnosed with minimal change disease (p=0.03, 0.02 and 0.002, respectively). ScRNA-seq analysis revealed decreased gene expression of TIM3 on T lymphocytes in DKD compared to control. And one of TIM-3's main ligands, Galectin-9 on immune cells showed a decreasing trend in gene expression as kidney damage worsened.

**Conclusion:**

Our study underscores the potential protective role of TIM-3 on T lymphocytes in attenuating the progression of DKD and suggests that monitoring circulating CD3+TIM3+ T cells may serve as a viable strategy for identifying DKD patients at heightened risk of disease progression.

## Introduction

In the context of type 2 diabetes mellitus (T2DM), the adaptive immune system plays a significant role in driving systemic inflammation and promoting insulin resistance, ultimately leading to the development of diabetic kidney disease (DKD) (1, 2). T cells, which are affected by hyperglycemia in various ways (including recruitment, activation, differentiation, maturation, and cytokine expression profile) (3, 4), are associated with both systemic and the local activation of inflammatory contributing to DKD progression. Specifically, in patients with DKD, the influx and accumulation of T cells in the juxtamedullary region of the glomerulus aggravates diabetic nephropathy and is associated with factors such as albumin excretion rate and filtration area (5–7). Moreover, T2DM patients with comorbid nephropathy often exhibit an imbalance in peripheral T cell subsets, with greater severity seen in those with DKD. This imbalance is characterized by polarization toward pro-inflammatory T cell subsets, such as the elevation of circulating CD8+ T lymphocytes (8) and T helper 17 (Th17) proportion, and the reduction of anti-inflammatory regulatory T cells (Tregs) proportion (9), indicating abnormal autoimmune regulation in DKD patients.

T-cell responses are strictly regulated to avoid sustained inflammation. They can upregulate a repertoire of immune checkpoint proteins/receptors on their surface, which upon binding with their respective ligands, activate negative regulatory signaling pathways within the cell. This effect reduces the production of pro-inflammatory cytokines and promotes the secretion of regulatory cytokines, resulting in the inhibition of effector T cell proliferation and reduction of inflammation (10). Notable examples of immune checkpoint molecules include programmed cell death protein 1 (PD-1) and its ligand (PD-L1), cytotoxic T-lymphocyte-associated protein 4 (CTLA-4), T cell immunoglobulin and mucin domain containing-3 (TIM-3), lymphocyte-activation gene 3 (LAG3), among others. A recent study utilizing mass cytometry technology examined peripheral blood mononuclear cell samples from six early-stage DKD patients and seven T2DM patients without kidney disease (11). The visualized results revealed a significant increase in the CD4 effecter memory T cells subgroup in the T2DM-DKD group accompanied by upregulation of CTLA-4 and PD-1 compared to T2DM without kidney involvement. In the streptozotoci-induced diabetic mouse model, blocking T-cell activation by soluble CTLA4-Fc fusion protein reduced the number of renal T-cells and albuminuria (12). These results indicated the potential role of immune checkpoint molecules in the development of DKD.

We hypothesized that reduced expression of immune checkpoint molecules on the T lymphocytes may have a deleterious effect on anti-inflammation signal, culminating in heightened inflammation and exacerbating the pathogenesis of DKD. Therefore, this study investigated the expression of immune checkpoint molecules on T lymphocytes in peripheral blood and kidney tissue, and analyzed their relationship with clinical parameters and renal pathology in DKD patients.

## Methods

### Ethics statement and study subjects

The ethical considerations and selection of participants were carefully considered in this study. The human research ethics committees at the Second Xiangya Hospital of Central South University reviewed and approved the human study protocol, which adhered to the World Medical Association Declaration of Helsinki. All study participants provided written informed consent and their demographic and clinical data were collected upon admission. In order to be eligible for inclusion in the study, for DKD group, participants had to be between 18 and 70 years old and have a diagnosis of type 2 diabetes, as defined by fasting glucose levels of 126 mg/dl or higher, random glucose levels of 200 mg/dl or higher, or use of insulin or anti-diabetes medication, as well as the presence of DKD, which was diagnosed in accordance with KDOQI clinical practice guidelines. Control samples were obtained from patients diagnosed with minimal change disease (MCD). A total of 360 DKD and 6 MCD subjects admitted between January 2018 and December 2021 were enrolled in the study.

### Flow cytometry

As previously described (13), single-cell suspension was immediately fixed upon isolation and stained for 30 min at 4°C with conjugated antibodies of the following specificities: CD45-PerCP, CD3-FITC, CD4-APC, CD8-PE, CD25-APC/Cy7, LAG3-APC, TIM-3-PE, CTLA-PerCP. Isotype and fluorescence-minus-one controls were used to set positive thresholds. Data acquisition and analysis were performed with a BD FACSCalibur and FlowJo software (Three Star).

### Histologic analysis

The renal specimens from 49 DKD patients without infection and other glomerular nephropathy were processed for immunofluorescence staining, light microscopy, and electron microscopy. Kidney sections were stained for antibody against CD3 (Abcam,1:400) and a secondary antibody Alexa Fluoro 488 (Abcam, 1:400), and antibody against TIM-3 (Abcam, 1:400) and a secondary antibody Alexa Fluoro 594 (Abcam, 1:400). The total number of CD3+ or TIM-3+ or CD3+TIM-3+ interstitial mononuclear cells within histological sections was quantified manually in all the fields.

### Single-cell RNA sequencing (scRNA-seq) acquisition

ScRNA-seq datasets were obtained from the Gene Expression Omnibus (GEO) repository, accessible at https://www.ncbi.nlm.nih.gov/gds/ (accession number: GSE184652), as documented in the publication (14) with PMID: 35709763. These datasets originated from two DKD murine models: the homozygous Lepr knockout (db/db) mouse model and the ReninAAV db/db uninephrectomy model. Specimens of later model were collected at two time points: 2 days and 2 weeks post-sacrifice. Notably, the ReninAAV db/db uninephrectomy model involved the induction of advanced DKD via adeno-associated virus-mediated delivery of renin to uninephrectomized db/db mice.

### Quality control, dimension reduction and clustering

The scRNA-seq data was imported into R (v4.3.3) using the *Read10X* function from the Seurat package (v4.4.0), which was used for all downstream analyses. An initial Seurat object was created using *CreateSeuratObject* to contain the raw data. Quality control was performed to retain only cells with >2000 UMIs (nCount_RNA) and mitochondrial content <5% (mitoRatio). Principal component analysis (PCA) was applied for dimensionality reduction. The determination of the optimal number of principal components (PCs) capturing maximal variance was facilitated by the elbow plot generated through *ElbowPlot*. Clustering of the integrated data was performed using *FindClusters* at a critical resolution parameter. The *Clustree* function was first utilized to examine relationships between resolutions, and 0.8 was selected for this study.

### Differential expression analysis

Differentially expressed genes (DEGs) between clusters were identified using the *FindAllMarkers* function with the default Wilcoxon rank sum test and a maximum adjusted p-value of 0.05. Cell classes were annotated based on the well-known kidney cell markers. The major 16 cell types observed and the immune cells were further clustering into B cells, T cells, dendritic cells and macrophages. The annotations were carefully checked for accuracy before visualizing clustering through uniform manifold approximation and projection (UMAP).

### Statistical analysis

Statistical analysis was performed using was performed using JMP (SAS Institute, Inc., Cary, NC, USA). Continuous variables are expressed as mean ± SD, and skewed variables as median (25th–75th percentile). Student-t test was used to compare continuous variables between the groups. For data that did not show a Gaussian distribution, comparisons were performed using Wilcoxon test. Regressions were calculated by the least-squares fit in correlation analysis. Univariate and multivariate analysis with logistic regression model was used to determine the potential correlation between interstitial infiltration equal or greater than 50% in renal biopsy and clinical characteristics. All tests were two-tailed, and p value <0.05 was considered to be statistically significant.

## Results

### T lymphocyte subset proportions in DKD patients

The present study analyzed a cohort of 360 DKD patients, and the results are presented in **Table 1**. Among these individuals, 63 (17.5%) exhibited concomitant glomerulonephritis (GN), including membranous nephropathy, IgA nephropathy, and focal segmental glomerulosclerosis. Additionally, 95 (26.4%) displayed manifestations of diabetic retinopathy (DR), while 62 (17.2%) suffered from diabetic peripheral neuropathy (DPN). Notably, 131 (36.4%) patients presented with bacterial or fungal infections affecting the respiratory, digestive, or urinary tracts. In terms of albuminuria levels, 6 (2.1%) of the cases exhibited normoalbuminuria (<30mg/day), 73 (25.3%) displayed microalbuminuria (30–300mg/day), and 210 (72.7%) demonstrated macroalbuminuria (>300mg/day). The present study identifies glomerular hematuria, characterized by an elevation of urinary red blood cell (RBC) counts beyond the threshold of 25/μl and a prevalence of dysmorphic RBCs of at least 80% among excreted RBCs, in 106 (39.8%) of the patients.

**Table 1:** Characteristics of the DKD cohort in the study.

Clinical variables	N=360
Age, year	56 ± 13
Sex (F/M)	117/243(32.5%/67.5%)
CKD stage	
1	34(9.4%)
2	32(8.9%)
3	76(21.1%)
4	76(21.1%)
5	142(39.4%)
Combined with other glomerular nephropathy, n(%)	63(17.5%)
Infection n(%)	131(36.4%)
Diabetic retinopathy, n(%)	95(26.4%)
Diabetic Peripheral Neuropathy, n(%)	62(17.2%)
Serum creatinine, μmoI/L	212.6(104.3,473.3)
eGFR, ml/min per 1.73 m2	22.2(9.4,51.1)
Albuminuria*	
Normo	6(2.1%)
Micro	73(25.3%)
Macro	210(72.7%)
Urinary red blood cell **/ul	18.9(7.6,65.8)

* n=289, ** n=266.

**Figure 1:**
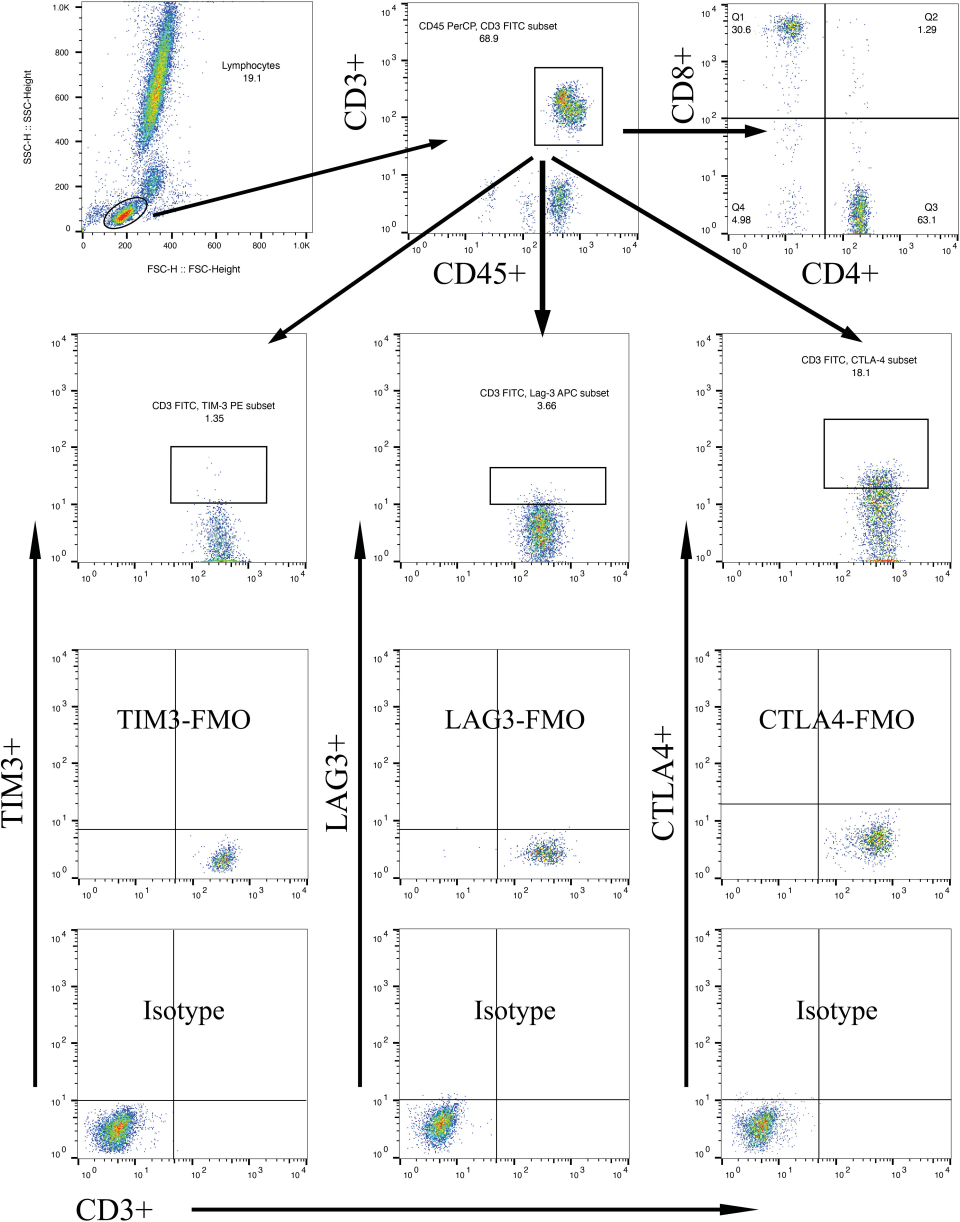
Gating strategies to discriminate various T cell subpopulations using multiparametric flow cytometry, with markers indicated on the x and y axes. Lymphocytes were gated based on forward scatter/side scatter dot plot. From the lymphocyte population, T lymphocytes were gated using surface markers CD45 and CD3. Subsequently, CD4+ T cells and CD8+ T cells were gated using their respective surface markers. T cell subpopulations within CD3+ lymphocytes were further gated based on TIM-3, LAG-3, or CTLA-4 expression.

**Figure 2:**
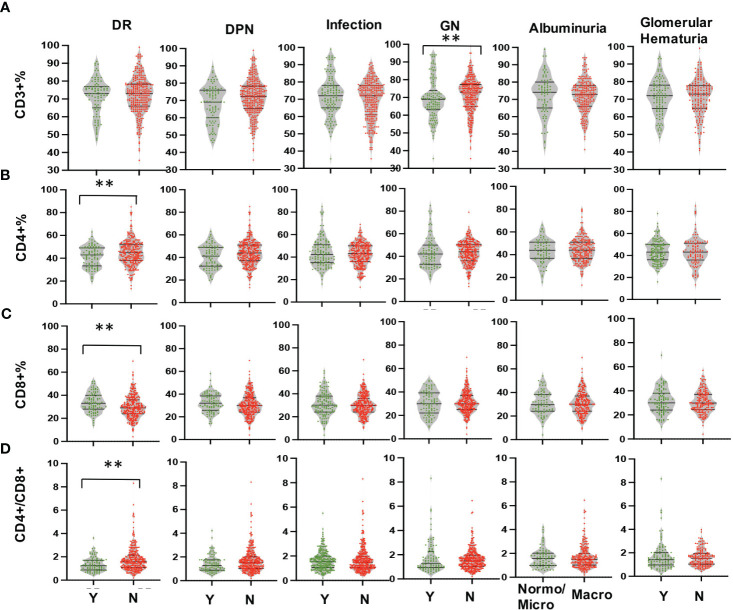
Comparison in proportions of the peripheral T lymphocyte subset [CD3+ **(A)**, CD4+ **(B)**, CD8+ **(C)**, CD4/CD8 **(D)**] among CD45+ lymphocytes in DKD patients with or without DR, DPN, infection, other GN, and with different degrees of albuminuria or hematuria. **p<0.01. DKD, diabetic kidney disease; DR, diabetic retinopathy; DPN, diabetic peripheral neuropathy; GN, glomerular nephropathy.

**Figure 3:**
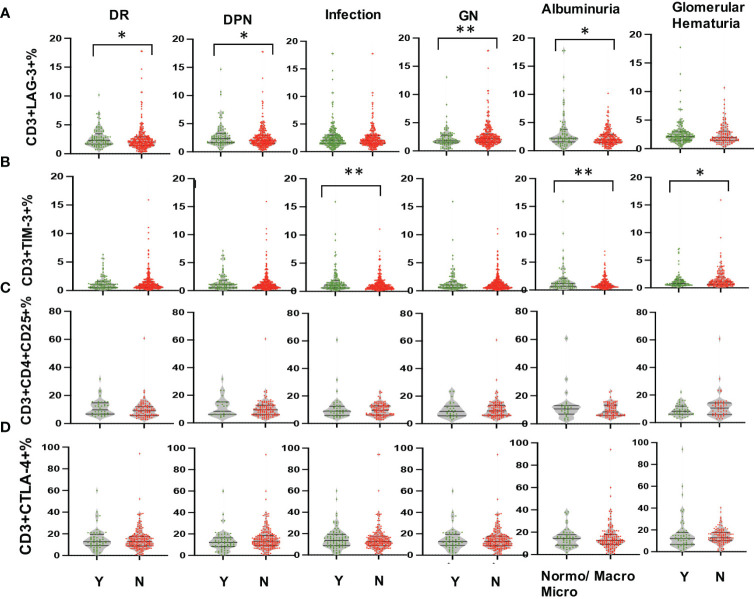
Comparison in proportions of the peripheral T lymphocyte subset [CD3+LAG-3+ **(A)**, CD3+TIM-3+ **(B)**, CD3+CD4+CD25+ **(C)**, CD3+CTLA-4+ **(D)** among CD45+ lymphocytes in DKD patients with or without DR, DPN, infection, other GN, and with different degrees of albuminuria or hematuria. *p<0.05, **p<0.01. DKD, diabetic kidney disease; DR, diabetic retinopathy; DPN, diabetic peripheral neuropathy; GN, glomerular nephropathy.

Multi-parameter flow cytometry was utilized to assess the proportional changes in circulating CD3+, CD4+, CD8+, CD3+TIM-3+, CD3+LAG3+, and CD3+CTLA-4+ T cell subsets across all enrolled patients. The flow cytometry gating strategy was illustrated in **Figure 1**. Subsequently, we analyzed the alterations in T lymphocyte subset proportions in the peripheral blood of DKD patients with different comorbidities or varying degrees of proteinuria or hematuria, as indicated in **Figures 2**, **3**. The results showed that patients with DR had a lower proportion of CD4+ and CD3+LAG-3+ T cells (p=0.009 and 0.023, respectively), and a higher proportion of CD8+ cells (p<0.0001) compared to those without DR (**Figures 2**, **3A**). DPN was solely associated with lower proportion of CD3+LAG-3+ T cells (**Figure 3A**, p=0.034). Coexistence of other glomerulonephritis was associated with lower proportion of CD3+ and CD3+ LAG-3+ T cells (**Figures 2A**, **3A**, p=0.003 and 0.004, respectively). Patients with infections had an increased proportion of CD3+TIM-3+ T cells compared with controls (**Figure 3B**, p=0.01), while those with macroalbuminuria or glomerular hematuria had a decreased proportion of CD3+TIM-3+ T cells compared with controls (**Figure 3B**, p=0.008 and 0.02, respectively).

### Proportion of peripheral CD3+TIM-3+T cells correlates with interstitial infiltration

**Figure 4:**
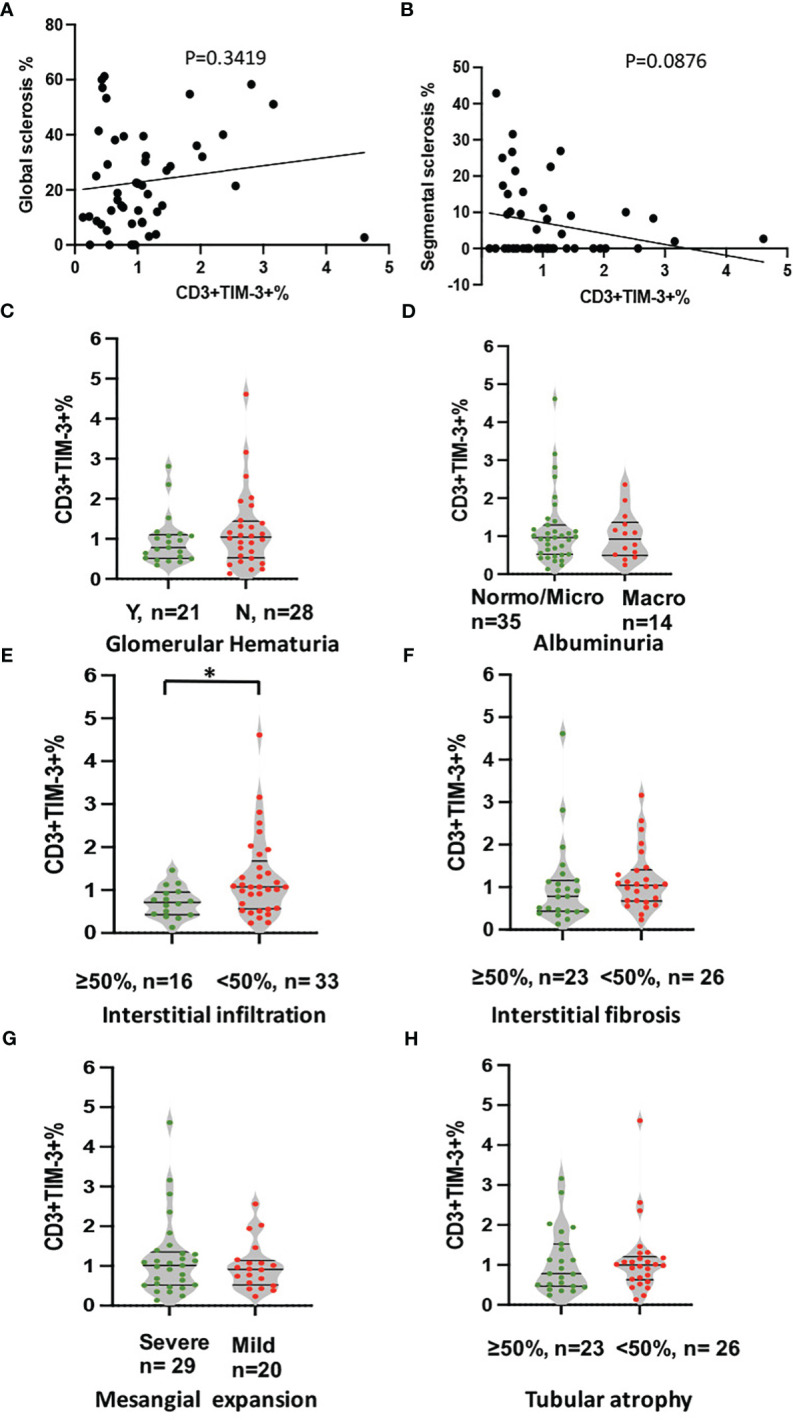
Analysis of correlation between expression of TIM-3 in peripheral T cells and pathological and clinical parameters. The analyses included **(A)** global sclerosis, **(B)** segmental sclerosis, **(C)** glomerular hematuria, **(D)** albuminuria, **(E)** interstitial in filtration, **(F)** IFTA, **(G)** Mesangial expansion and **(H)** Tubular atrophy. *p<0.05.

To avoid confounding factors, this study exclusively examined renal specimens obtained from 49 DKD patients, devoid of co-occurring instances of infection or other glomerular nephropathy. As it shown in **Figure 4**, the expression of Tim-3 in peripheral T cells was no longer higher in patients with macroalbuminuria or glomerular hematuria, possibly due to the smaller sample size (**Figures 4C, D**). However, a reduced expression of Tim-3 was observed in peripheral T lymphocytes of DKD patients who exhibited greater infiltration (>50%) of interstitial mononuclear cells in their renal tissue (**Figure 4E**, p=0.019).

The statistical analysis in **Table 2** revealed that glomerular hematuria (**Table 2**, OR: 5.060, 95%CI: 1.390,18.415, p=0.014) and the proportion of CD3+TIM-3+T cells (**Table 2**, OR: 0.203, 95%CI: 0.047,0.871, p=0.032) were significantly associated with severe interstitial infiltration as demonstrated by the univariate logistic regression analysis. Moreover, the multivariate logistic regression analysis, with glomerular hematuria and the proportion of CD3+TIM-3+T cells as covariates, still highlighted a robust correlation between the proportion of CD3+TIM-3+ T cells and severe interstitial infiltration (**Table 2**, OR: 0.193, 95%CI: 0.040,0.926, p=0.04).

**Table 2:** Univariate and multivariate analysis between severe renal interstitial infiltration and clinical parameters in DKD patients.

	Univariate logistic regression	P value
	OR (95%CI)	
Sex (Female)	0.286(0.055,1.486)	0.137
Age, per 1-year increase	1.039(0.980,1.101)	0.203
White blood cell, per SD	1.134(0.952,1.350)	0.160
Hemoglobin, per SD	0.998(0.976,1.021)	0.872
Albumin, per SD	0.986(0.909,1.069)	0.730
Triglycerides, per SD	1.428(0.986,2.068)	0.060
Total cholesterol, per SD	0.914(0.716,1.166)	0.469
Low-density lipoprotein, per SD	0.841(0.602,1.174)	0.301
High-density lipoprotein, per SD	0.818(0.354,1.891)	0.639
Serum creatine, per SD	0.994(0.982,1.007)	0.391
Estimated glomerular filtration rate, per SD	1.002(0.984,1.021)	0.830
Macro-albuminuria	1.304(0.337,5.048)	0.700
Glomerular hematuria	5.060(1.390,18.415)	**0.014**
CD3+, per SD	0.967(0.889,1.052)	0.437
CD4+, per SD	0.986(0.919,1.057)	0.685
CD8+, per SD	0.979(0.917,1.045)	0.515
CD4/CD8, per SD	0.954(0.572,1.592)	0.857
CD3+TIM-3+, per SD	0.203(0.047,0.871)	**0.032**
CD3+LAG-3+, per SD	0.520(0.260,1.040	0.065
CD3+CD4+CD25+, per SD	0.788(0.602,1.030)	0.081
CD3+CTLA-4+, per SD	0.889(0.749,1.054)	0.177
	Multivariate logistic regression	P value
	OR (95%CI)	
Glomerular hematuria	5.096(1.264,20.544)	**0.022**
CD3+TIM-3+, per SD	0.192(0.040,0.926)	**0.040**

Bolded value: P value < 0.05.

### Augmented Tim-3 expression on renal interstitial mononuclear cells in DKD

**Figure 5:**
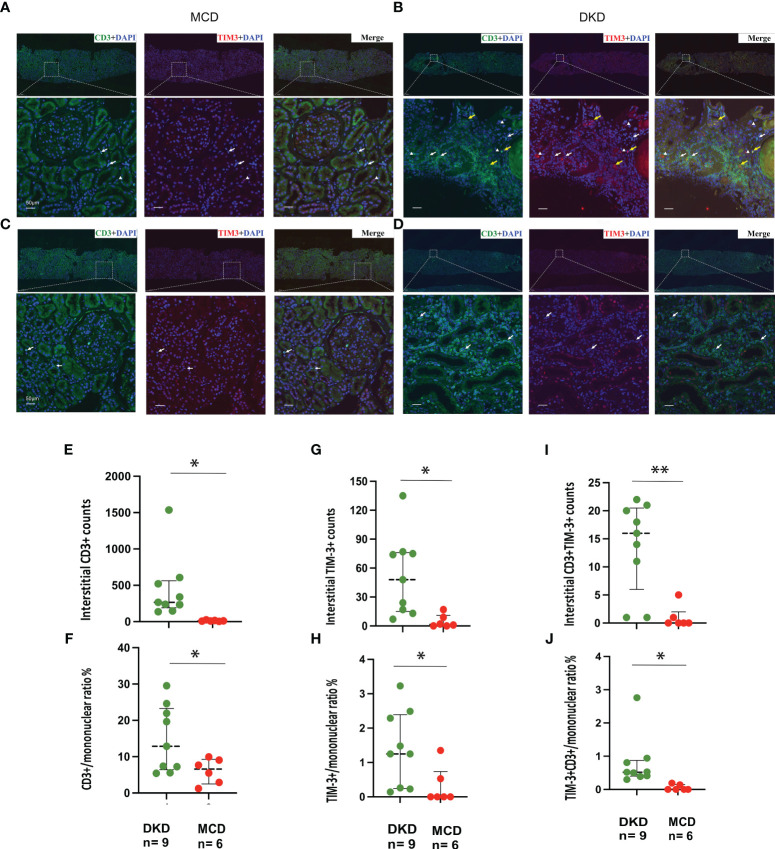
TIM-3 expression on renal intersitial mononuclear cells in DKD and MCD. **(A–D)** Representative immunofluorescence staining images (× 40) of kidney CD3+ (green) and TIM-3+ (red) interstitial cells from 2 MCD patients and 2 DKD patients. Double staining is in yellow. Yellow arrow, CD3+TIM-3+; white arrow, CD3+TIM-3-; white triangle, CD3-TIM-3+. **(E, F)** The numbers of interstitial CD3+cells and the ratio of CD3+/mononuclear cells increased in DKD compared with MCD. **(G, H)** The numbers of interstitial TIM-3+cells and the ratio of TIM-3+/mononuclear cells increased in DKD compared with MCD. **(I, J)** The numbers of interstitial CD3+TIM-3+cells and the ratio of CD3+TIM-3+/mononuclear cells increased in DKD compared with MCD. *p<0.05, **p<0.01. DKD, diabetic kidney disease; MCD, minimal change disease.

Immunofluorescence staining for CD3 and TIM-3 was performed on kidney sections obtained from a total of 15 patients, comprising 9 DKD and 6 MCD cases (**Figure 5**). It was observed that patients with DKD were of an advanced age and presented with a significantly reduced eGFR (**Table 3**, p=0.023 and 0.022, respectively) compared with MCD patients. Additionally, the renal pathology of DKD patients exhibited a greater degree of glomerular sclerosis, interstitial infiltration, and mesangial expansion (**Table 3**, p=0.015, 0.025 and 0.003, respectively). As depicted in **Figure 5**, immunofluorescence assays revealed an increase in CD3+ and TIM-3+ interstitial cells in the kidneys of DKD patients as compared to MCD (p=0.03 and 0.02, respectively). Interstitial monocular cells in DKD also exhibited a higher degree of co-localization of TIM-3 and CD3+ (p=0.002). Moreover, the ratio of CD3+, TIM-3+, and CD3+TIM-3+ to mononuclear cells were all significantly increased in DKD compared with which in MCD (**Figure 5F**). In the renal interstitium of DKD patients depicted in **Figure 5D**, a notable infiltration of CD3+ mononuclear cells was observed, while TIM3+ cells were scarce, and the patients exhibited glomerular hematuria. Conversely, in the interstitium of DKD patients illustrated in **Figure 5B**, a higher abundance of CD3+TIM3+ cells was detected, and the patients did not present with glomerular hematuria.

**Table 3:** Laboratory data and pathological characteristics of DKD and MCD cohort.

Parameter	DKD(n=9)	MCD(n=6)	P value
Gender (Female), n (%)	2(33.3)	4(66.7)	0.085
Age, year	58 ± 7	36 ± 17	**0.023**
White blood cell, 10^9^/L	8.4 ± 2.6	7.4 ± 2.1	0.400
Hemoglobin, g/L	130.2 ± 28.0	127.6 ± 19.4	0.841
Albumin, g/L	27.3 ± 7.6	35.3 ± 11.0	0.159
Serum creatine, μmoI/L	90.7(65,3, 117.5)	66.7(58.1, 83.8)	0.195
eGFR, ml/min per 1.73 m^2^	66.8 ± 31.9	105.1 ± 24.7	**0.022**
Proteinuria, mg/24h	4202(1270, 5850)	3589(645, 10689)	0.556
Hematuria,/μl	41.6(26.3, 171.5)	41.6(4.5, 189.1)	0.637
Glomerular sclerosis, %	25.0(12.5, 39.7)	1.5(0, 8.2)	**0.015**
Interstitial fibrosis >25%, n (%)	7(77.8)	2(33.3)	0.150
Interstitial infiltration >50%, n (%)	4(44.4)	0(0)	**0.025**
Severe mesangial expansion, n (%)	6(55.7)	0(0)	**0.003**
Tubular atrophy >50%, n (%)	3(33.3)	0(0)	0.059

DKD, diabetic kidney disease; MCD, minimal change disease; eGFR, estimated glomerular filtration rate. Bolded value: P value < 0.05.

### Gene expression of Tim-3 and its ligands in DKD mouse model

**Figure 6:**
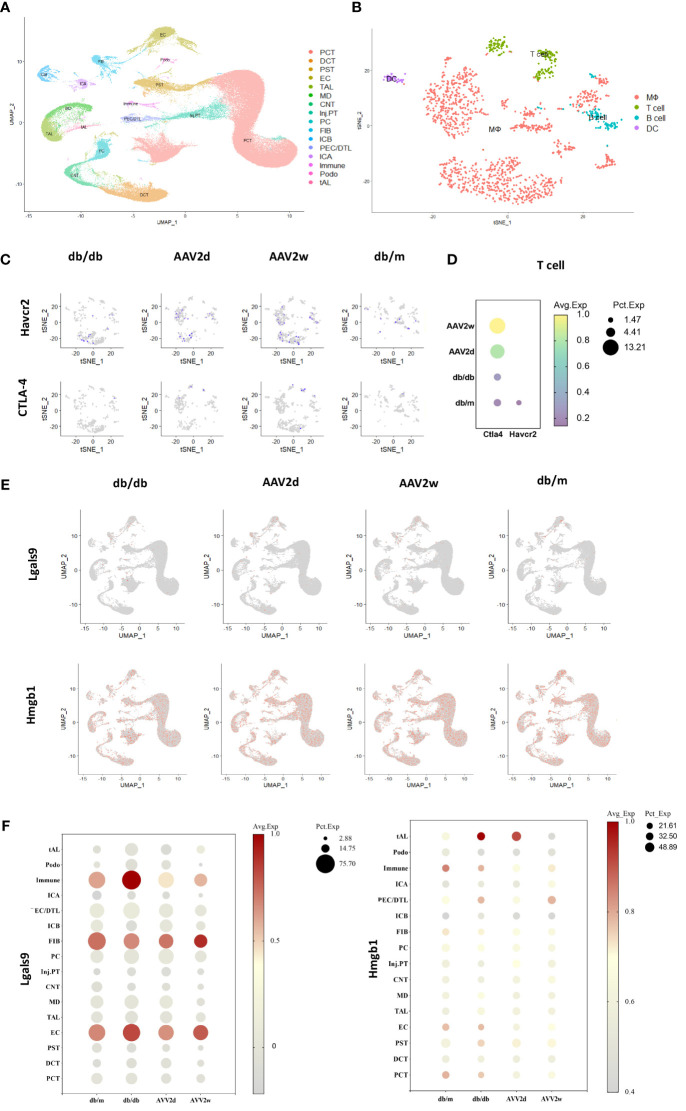
The single-cell analysis conducted on cells of 12 mouse kidneys from DKD(db/db), advanced DKD (db/db+renin 2d, db/db +renin 2w), and control (db/m) groups to assess the expression of CTLA-4, TIM-3 (Havcr2) and TIM-3’s ligands (Gal-9, Hmgb1). **(A)** Cells from total 12 mouse kidneys in 4 different groups are projected by uniform manifold approximation and projection (UMAP) plot. **(B)** Integrated clustering plot of mouse kidney immune cells. **(C)** Havcr2(TIM-3)+ and CTLA-4 cells in control (db/m), db/db, or db/db +Renin-AAV at 2 days and 2 weeks. **(D)** Expression of Havcr2(TIM-3) and Ctla4 in T cells across all the groups. **(E)** Lgals9(Gal-9)+ cells and Hmgb1 in control (db/m), db/db, or db/db +Renin-AAV at 2 days and 2 weeks. **(F)** Expression of Lgals9(Gal-9) and Hmgb1 in different cells across all the groups. PCT, Proximal Convoluted Tubule; PST, Proximal Straight Tubule; EC, endothelial cells; TAL, thick ascending limb; tAL, one of the TAL subclusters highly expressing Atp10b; MD, macular densa; CNT, connecting tubule; inj.PT, Injuried PT; PC, principal cells; FIB, Fibroblast; PECs, parietal epithelial cells; DCT, distal convoluted tubule; ICA, type A intercalated cells; ICB, type B intercalated cells; Podo, podocyte; DTL, descending thin limbs of Henle’s loop; MΦ, Macrophage; DC, dendritic cell.

We analyzed scRNA-seq data from the kidneys of DKD and severely affected DKD mouse model (**Figures 6A–C**). Our findings indicate a notable elevation in CTLA-4 gene expression within T cells concomitant with the exacerbation of DKD, whereas the gene expression of TIM-3 (Havcr2) was solely discernible in the control cohort (**Figure 6D**). Additionally, further analysis was conducted on the gene expression of TIM-3's main ligands, Galectin-9 (Gal-9, Lgals9), and high mobility group protein B-1 (Hmgb-1) (**Figures 6E, F**). Lgals9 was primarily expressed on immune cells, endothelial cells, and fibroblasts, with a decreasing trend in gene expression as kidney damage worsened. Hmgb-1 was predominantly expressed in thick ascending limb cells.

## Discussion

Dysfunction of T cell subgroups can disrupt cellular and humoral immune homeostasis, resulting in sustained inflammation that contributes to the progression of DKD (2). However, a comprehensive understanding of the specific regulatory mechanisms is lacking, and effective clinical interventions remain elusive. Recent investigations into immune checkpoint molecules have revealed their potential as agents for modulating T cell function. Immune checkpoint molecules not only exhibit a common inhibitory effect on T cell activation, but also activate downstream signal pathways that can exert synergistic regulation. These pathways include inhibition of effector T cell activation, promotion of Treg function, and reduction of innate immune cell inflammation activity (15). Our research has yielded novel insights into the role of Tim-3, an immune checkpoint molecule, in the progression of kidney damage in DKD. The primary observations revealed a close correlation between the expression of Tim-3 on peripheral T cells and the manifestation of renal inflammation, alongside interstitial ratio of Tim-3 to CD3 and diabetic nephropathy.

Tim-3 is a cell surface protein that is significantly expressed on various immune cells, and plays a crucial role in regulating essential biological processes of immune cells, such as cellular interactions, synthesis of effector proteins, proliferation, apoptosis, and phenotypic switching (16). The function of TIM-3 differs in the diverse cell types in which it is expressed, playing a crucial role in the development of autoimmune diseases, infections, cancers, transplant rejection, and chronic inflammation (17–20). In our study, the lower proportion of peripheral CD3+Tim-3+ cells from DKD patients has been associated with urine proteinuria and glomerular hematuria which is closely related to kidney inflammation (21). Furthermore, more interstitial mononuclear cells infiltration in diabetic kidney was associated with less circulating CD3+TIM-3+ cells.

We hypothesized that the observed downregulation of TIM-3 on peripheral T cells may contribute to the exacerbation of kidney damage in individuals with DKD. This may occur, in part, due to a decrease in the function of Tregs, which could have a role in regulating the progression of diabetes by suppressing the activation of effector T cells and exerting anti-inflammatory effects. Previous research has demonstrated that circulating CD4 CD25 Foxp3 Tregs are notably diminished in T2DM-DKD patients, and the ratio of proteinuria to Tregs is negatively correlated (22). Removing Tregs with anti-CD25 mAb exacerbates diabetes-related kidney damage in mice (23), while transferring Tregs has the opposite effect (23). It should be noted that in various diseases, a decrease in the secretion of IL-10 by Treg cells is linked to the downregulation of TIM-3 in Treg cells (24, 25). Further research is required to delineate if which subtype of peripheral T cells in DKD experiences changes in TIM-3.

This study delves deeper into the link between interstitial expression of TIM-3 and diabetic renal pathology. Results indicate that DKD patients exhibit greater renal inflammation compared with MCD patients, as evidenced by increased infiltration of interstitial mononuclear and CD3+ cells. Furthermore, greater infiltration of CD3+ TIM-3+ cells was observed in DKD patients, supporting the notion that dysfunction of T cell subgroups may contribute to renal inflammation and injury in DKD. Our scRNA-seq investigation unveiled the exclusive presence of TIM3 expression within T cells isolated from db/m mice, concomitant with a simultaneous escalation in CTLA4 expression levels in T cells, correlating with the exacerbation of renal impairment. These findings parallel human data (14, 26), wherein renal tissue from individuals afflicted with DKD exhibited a decrease in TIM3 expression alongside an elevation in CTLA4 expression in T cells compared to control counterparts. This observation hints at the plausible involvement of immune checkpoint molecule modulation within T lymphocytes in the pathogenesis of renal damage. Moreover, the regulatory role of TIM3 in T cells within the context of DKD remains underexplored. Blocking the TIM-3- Gal-9 pathway significantly reduces the regulatory function of Tregs (27), impeding anti-inflammatory pathways and potentially exacerbating the progression of the disease. The binding of TIM-3 and High mobility group box 1 on the surface of CD8 Treg cells can significantly inhibit effector T cells (28). Consequently, we scrutinized the expression patterns of its ligands in the renal tissues of DKD mice, revealing a noteworthy declining trajectory in expression, particularly evident in Gal-9, as renal injury progressed. The potential implication of the TIM-3-Gal-9 pathway in the progression of DKD necessitates further thorough examination.

On the contrary, heightened expression of TIM-3 has been observed on peripheral blood T lymphocytes in patients afflicted by combined infection and diabetic nephropathy. These outcomes align with prior research that posits an increase in TIM-3 expression in T cells following infection (29). Our prior investigations have demonstrated that CD3+TIM-3+ T cells may not play an anti-inflammatory role in the context of chronic infection, but instead, promote inflammation (30). Moreover, our research has revealed that patients with diabetes who have developed DR, DPN, or other glomerular nephropathy exhibit down-regulation of LAG-3 on peripheral T lymphocyte compared to controls. In Non-Obese Diabetic mice, the absence of LAG-3 is associated with a progression of diabetes (31). LAG-3 also plays a role in the proliferation and function of Tregs (32) and may be implicated in the development of other complications of diabetes or immune-mediated renal disease. Ascertaining the precise mechanism underlying this phenomenon will require further investigation.

Research also indicates that TIM-3 expression extends beyond T cells to include macrophages, a significant area of study within the TIM-3 field. In macrophages, TIM-3 induction has been found to suppress inflammation and promote tissue repair. It facilitates the polarization of M0 to an anti-inflammatory M2 phenotype via interactions with ligands like Galectin-9 (33). Additionally, TIM-3 reduces ROS production and pro-inflammatory cytokines IL-1β and IL-18 (34), aiding in inflammation control and tissue repair. On the contrary, it has also been reported that TIM-3 mediates podocyte damage via NF-κB/TNF-α pathway activation in macrophages in DKD (35). TIM-3 may also activate mast cells (36) and assist in dendritic cell antigen presentation (37). Given its diverse functions and context-dependent effects, understanding TIM3’s roles in DKD requires consideration of disease models, cell types, and receptors.

In conclusion, the present study demonstrates potential significance of TIM-3 on T lymphocytes in DKD progression. And the findings suggest that monitoring the CD3 + TIM-3 + T cells in the periphery may be useful in evaluating DKD patients who may be at an increased risk of progressive kidney inflammation. The study provides novel insights into the role of TIM-3 in DKD and potential therapeutic targets for future research. Further mechanistic studies, with a focus on the signaling pathways involved, are warranted to better understand the mechanisms underlying the observed TIM-3 and T cell responses during development of DKD.

## Data availability statement

The original contributions presented in the study are included in the article/supplementary material. Further inquiries can be directed to the corresponding authors.

## Ethics statement

The studies involving humans were approved by the human research ethics committees at the Second Xiangya Hospital of Central South University. The studies were conducted in accordance with the local legislation and institutional requirements. The participants provided their written informed consent to participate in this study.

## Author contributions

XC: Formal analysis, Writing – original draft, Writing – review & editing. RT: Writing – review & editing, Investigation. JZ: Data curation, Formal analysis, Investigation, Writing – review & editing. LZe: Writing – review & editing, Formal analysis. LZh: Writing – review & editing, Validation. ZL: Investigation, Writing – review & editing, Data curation. DY: Writing – review & editing, Investigation. MZ: Writing – review & editing, Investigation, Methodology. XZ: Writing – review & editing, Data curation. AC: Writing – review & editing, Formal analysis. HL: Conceptualization, Writing – review & editing, Supervision. HC: Conceptualization, Supervision, Writing – review & editing. GC: Conceptualization, Funding acquisition, Writing – review & editing.
